# A critical evaluation of methods to interpret drug combinations

**DOI:** 10.1038/s41598-020-61923-1

**Published:** 2020-03-20

**Authors:** Nathaniel R. Twarog, Michele Connelly, Anang A. Shelat

**Affiliations:** Department of Chemical Biology and Therapeutics, St. Jude Children’s Research Hospital, 262 Danny Thomas Place, Memphis, TN 38105 USA

**Keywords:** Computational biology and bioinformatics, Drug discovery

## Abstract

Combination therapy is increasingly central to modern medicine. Yet reliable analysis of combination studies remains an open challenge. Previous work suggests that common methods of combination analysis are too susceptible to noise to support robust scientific conclusions. In this paper, we use simulated and real-world combination datasets to demonstrate that traditional index methods are unstable and biased by pharmacological and experimental conditions, whereas response-surface approaches such as the BRAID method are more consistent and unbiased. Using a publicly-available data set, we show that BRAID more accurately captures variations in compound mechanism of action, and is therefore better able to discriminate between synergistic, antagonistic, and additive interactions. Finally, we applied BRAID analysis to identify a clear pattern of consistently enhanced AKT sensitivity in a subset of cancer cell lines, and a far richer array of PARP inhibitor combination therapies for BRCA1-deficient cancers than would be identified by traditional synergy analysis.

## Introduction

Few ideas are more central to modern medicine than combination therapy. Multi-agent therapies drive therapeutic development and treatment in a wide range of diseases, including cancer^1–4^, as well as infectious diseases such as AIDS^5–7^, tuberculosis^8,9^, and malaria^10,11^. The advantages of combination therapy are clear: by attacking a disease through multiple mechanisms, the potency and efficacy of treatment can be increased while maintaining or even reducing negative side effects. Further, use of combination therapy leverages the development of otherwise ineffective drugs, and allows for repurposing of existing, clinically-approved agents, greatly accelerating the discovery and development of new therapies.

Combination therapeutic development, however, introduces its own challenges. As the number of individual agents grows, the space of potential combination therapies undergoes a combinatorial explosion. It is infeasible to evaluate all possible combination therapies in a clinical setting, or even in *in vivo* preclinical studies. Rational and robust prioritization of combination therapies through *in vitro* evaluation is therefore indispensable to successful therapeutic development. In addition, evaluation of combination therapies *in vitro* can inform the understanding of genetic and biomolecular factors that drive the efficacy of a particular combination, allowing for more efficient discovery of new combination therapies^12,13^. Unfortunately, quantitative evaluation of combined action is notoriously challenging, both experimentally and analytically, requiring significantly more measurements than single-agent dose-response evaluation, and with no widely accepted general model analogous to the Hill equation for single agents.

Given these challenges, many experiments evaluating combined action focus on the identification of *synergy*, in which a combination is more potent than individual activity would suggest, and *antagonism*, in which the combination is less potent than expected. This emphasis on the type of interaction is based on the relative simplicity of extracting and evaluating a single number describing a combination, and on the assumption that synergistic combinations are more promising than antagonistic or non-interacting combinations. The high demand for such methods explain why the two papers describing the two most popular combination evaluation methods – the Combination Index^14^ (‘CI’) and Bliss independence^15^ – have over 5100 citations between them as of this writing (CI also appears under other names, including the interaction index or sum of FICs^16^). These methods are extraordinarily common, being used in hundreds of papers each year; yet we have been unable to find a systematic evaluation of these methods under a range of simulated and real-world experimental and pharmacological conditions.

In a previous paper, we reported that one of these methods, CI, exhibited significant, variable instability in response to a range of conditions; we also derived a response surface model, BRAID, which exhibits far greater statistical stability^17^. In this paper, we investigate this index instability further, along with the Bliss-independence based *Bliss volume* method (‘Bliss’). Using a combination of simulated response surfaces and real-world large-scale combination data, the Merck OncoPolyPharmacology Screen (‘OPPS’)^18^, we show that these index-based methods are not only unstable, but biased towards synergy or antagonism according to experimental and pharmacological conditions. Simulated surfaces begin with the simplifying assumption of Loewe additivity, which we extend to combinations with differing maximal effects with a model we call *asymptotic Loewe additivity* (see Methods). Here, we also show that the previously introduced BRAID model of combined action is more stable and exhibits less bias across all simulated conditions tested.

A statistically robust metric, however, must still be shown to be scientifically informative. In the absence of a clear ground truth on general compound interaction, we evaluate these metrics with the following hypothesis: a metric which accurately describes how a compound interacts with another compound (synergy, antagonism, or additivity) should carry information about that compound’s mechanism of action. Using OPPS, which reported 22,410 combinations from 38 compounds across 39 cell lines, we perform hierarchical clustering based on all three combination metrics to test their agreement with reported compound mechanism of action. The BRAID interaction parameter κ outperforms both CI and Bliss, and the index of achievable efficacy (IAE), a BRAID-based measure that incorporates both interaction and individual potency, outperforms all methods.

We use BRAID to explore OPPS further. Using the IAE, we identify a subset of cancer cell lines which are selectively sensitive to AKT inhibition in a manner that is independent of sensitivity to inhibition of the PI3K/mTOR pathway. We then interrogate the relationship between the loss of function of BRCA1 – a tumor suppressor gene critical to homologous recombination repair (HRR)^19^ – and the combination efficacy of the PARP inhibitor Niraparib^20^. This set of experiments is of particular interest due to ongoing investigations of PARP inhibitor therapies in cancers exhibiting deficiencies in HRR^21^. We show that combinations involving Niraparib exhibit a range of changes in synergy in response to a loss of BRCA1 function; more importantly, these changes in synergy are largely uncorrelated with changes in combined efficacy as measured by IAE, revealing several combinations that are selectively more potent in a BRCA1 deficient cell-line despite a relative lack of synergy. These results suggest that prioritizing combinations based on the degree of synergy may fail to identify promising combinations that exhibit specific efficacy without any corresponding change in interaction, a distinction we refer to as *synergy-driven* vs. *potency-driven* efficacy. Finally, we show that combination behavior can be used to map different cell lines along a dimension corresponding to the phenotype of BRCA1 deficiency, identifying several cell lines with an even more pronounced sensitivity to Niraparib combinations, suggesting a “hyper-deficient” phenotype among the cell lines tested.

## Results

### Bliss and CI are biased by the shape of the individual dose-response curves

Our general approach to evaluate the Bliss and CI methods was to simulate drug combinations according to the principle of Loewe additivity, one of the most commonly applied models of non-interaction^22^. Loewe additivity is popular for modeling pharmacological interactions in particular because it posits that a combination of a compound with itself, or two compounds that differ by potency but otherwise behave the same, must be additive.

**Figure 1:**
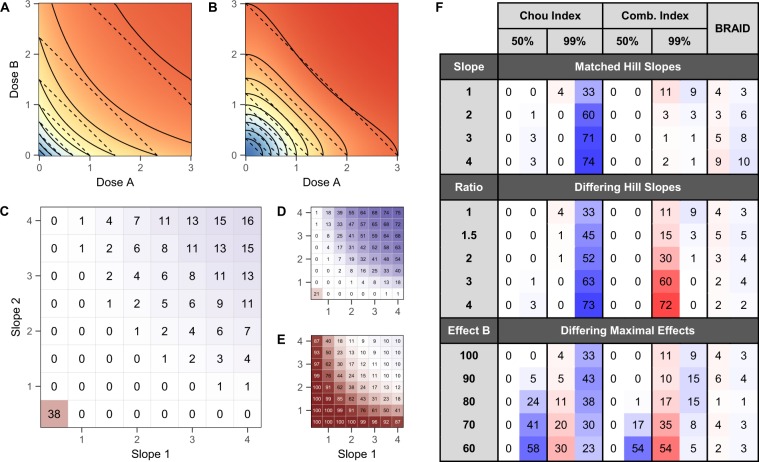
(**A**) Plot comparing the response surface shape of a Bliss independent surface (solid contours) with a Loewe additive surface (dotted contours) between two drugs with low Hill slopes of 1. **(B)** Plot comparing Bliss independence and Loewe additivity between two drugs with higher Hill slopes of 2. **(C)** Percentage of simulated Bliss screens in which each asymptotically additive combination of compounds was judged non-additive. Red cells indicate combination was judged antagonistic more than synergistic; blue indicate combination was judged synergistic more than antagonistic. **(D)** Percentage of non-additive judgments in Bliss screens in which the thresholds for synergy and antagonism were set using only low-Hill-slope drugs. **(E)** Percentage of non-additive judgments in Bliss screens in which the thresholds for synergy and antagonism were set using only high-Hill-slope drugs. **(F)** Percentage of simulated surfaces judged to be antagonistic (red) or synergistic (blue) in a range of pharmacological conditions based on the Chou Index (at the 50% and 99% effect level), the Combination Index (at the 50% and 99% effect level), and the BRAID response surface method. “Matched Hill Slopes” refer to simulations in which the Hill slopes of simulated drugs were equal to one another, but varied from the default value of 1; “Differing Hill Slopes” refer to simulations in which the two slopes diverged such they differed by the specified ratio; and “Differing Maximal Effects” refer to simulations in which one of the simulated drugs reached only a partial effect (between 60% and 90%) even at the highest concentrations.

Bliss measures a surface’s deviation from Bliss independence, which assumes that compound effects are probabilistically independent events: the probability that a cell will survive some dose of drug 1 and some dose of drug 2 is the product of the probabilities that the cell would survive either dose alone. However, Bliss failed to satisfy Loewe additivity in a Hill slope dependent manner. As shown in Fig. 1A, a Loewe additive surface combining two compounds with low Hill slopes appeared less potent in combination than Bliss, and would thus be judged antagonistic by the Bliss method. For combinations with higher Hill slopes the effect was reversed (Fig. 1B). Because Bliss presents no clear rule for selecting thresholds for synergy and antagonism, thresholds are often derived empirically by defining a statistical metric (e.g., one standard deviation) based on the range of Bliss values observed from self vs. self combinations in a study. However, as we just noted, these self vs. self thresholds vary considerably according to Hill slope. To better illustrate this point, we simulated triplicate Bliss interaction surfaces for eight self vs. self drug combinations with Hill slopes ranging from 0.5 to 4 in increments of 0.5. The mean percent deviation in Bliss was negative for Hill slopes equal to 0.5 and 1.0 (−10.30% and −2.87%, respectively), then switched positive, and reached 2.78% for Hill slope equal to 4.0 (Table S1).

To elucidate how the Hill slope affects the interpretation of drug combinations using Bliss, we simulated 1,000 all vs. all combinations of eight drugs with varying Hill slopes (from 0.5 to 4) according to Loewe additivity. Each combination in a screen was tested in a checkerboard layout, with Gaussian noise with a standard deviation of 5% effect added to all measurements. The cutoffs to designate synergy and antagonism were defined as Bliss deviations above or below, respectively, the 95% confidence interval of the 24 Bliss values from the self vs. self simulation described above. As noted earlier, this calibration of Bliss values is common in real-world studies and gives this method the best chance of identifying variations in interaction among the screened compounds by attempting to account for Hill slope dependency. Figure 1C depicts the results of these simulated screens: nearly 40% of simulated combinations where both drugs have Hill slopes less than 1 were judged antagonistic, and combinations where both slopes were greater than 3 were judged synergistic 13–16% of the time. Judgments of synergy and antagonism by Bliss are so dominated by variations in individual dose-response behavior that genuine synergy or antagonism would likely be missed altogether.

Finally, because we used a wide range of Hill slopes to define the range of self vs. self behaviors and determine threshold, we asked what happens when the set of dose-response shapes used to set the thresholds does not fully encompass the set of shapes found in the final dataset. To examine this, we re-ran the 1,000 simulations, but in each simulated screen used only the compounds with the lowest four Hill slopes (ranging from 0.5 to 2) to estimate thresholds for synergy and antagonism. The result was a slightly reduced – but still significant – bias towards antagonism for the lowest slope combinations, and a drastic bias towards synergy at higher Hill slopes (Fig. 1D): over 50% of additive combinations were judged synergistic by Bliss when the product of Hill slopes was greater than 7. Performing the corresponding simulation using only higher Hill slopes (ranging from 2.5 to 4) to set thresholds results in an overwhelming bias towards antagonism, with over 90% of additive combinations judged antagonistic when the product of Hill slopes is less than 2 (Fig. 1E).

CI, by contrast, does measure deviations from Loewe additivity; for a given constant-ratio combination of two drugs, the CI at a particular effect level (e.g. 90% killing) is the ratio of the combined concentration that produces that effect and the expected concentration that would produce that effect according to Loewe additivity. Figure 1F summarizes the results of simulated combination experiments using two versions of the CI, one employing the median effect method of dose-response analysis^23^, and the other using a more robust least-squares optimization. These runs simulated 1,000 individual checkerboard experiments on asymptotically Loewe additive combinations, using a consistent cutoff of 0.5 for synergy and 2 for antagonism. One limitation of the CI method is that is requires selecting a particular dose pair or dose ratio and an effect level at which to be calculated; different selections will result in different values, even in ideal circumstances. To address this, we calculated the value of CI for seven dose ratios and two effect levels, 50% and 99%. Though the use of a more robust fitting improved estimates, particularly at high effect levels, both methods exhibited large variations in stability and bias, with the least-squares method particularly biased towards antagonism at high effect levels when the slopes of the two dose-response curves differ, and biased in opposite directions at different levels when applied to combinations containing a drug with incomplete effect. For comparison, we have also included the rates at which the BRAID model falsely posited synergy or antagonism based on 95% confidence intervals. Though such errors do occur, they are symmetrically distributed, and never above 10% across all conditions tested.

### Evaluating metric bias using the OPPS

**Figure 2:**
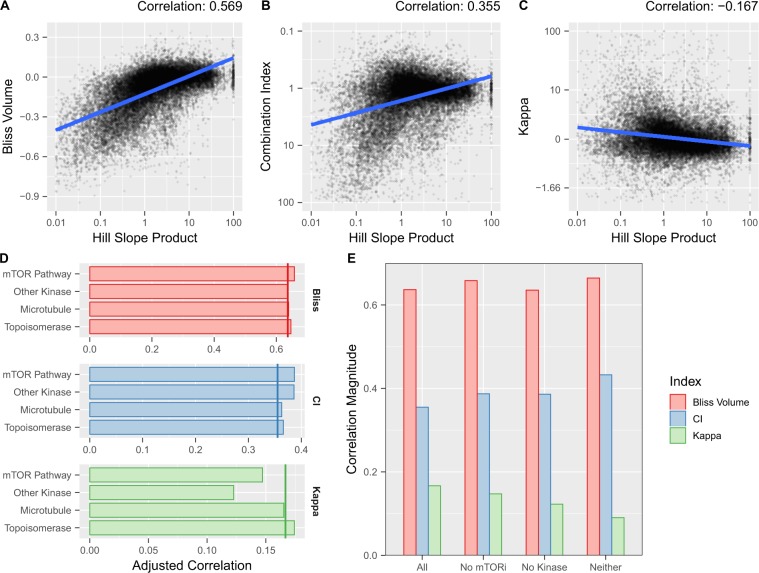
(**A–C**) Plot of the product of the best fit Hill slopes and calculated Bliss, CI, and BRAID κ for each combination of drugs in each cell line from the OPSS. The product of the best-fit individual dose response Hill slopes is plotted on a logarithmic scale; the blue line represents the best linear fit to the data. For CI, the Y-axis has been inverted so that values representing synergy are higher, to maintain visual consistency with figures (**A**,**C**). **(D)** Magnitude of correlation between Hill slope product and all three interaction metrics when drugs from each of the four largest mechanistic classes are removed from the data. No class significantly reduces correlation for Bliss or CI, but two reduce the correlation for κ. **(E)** Effect on correlation magnitude of removing PI3K/AKT/mTOR pathway inhibitors, Other Kinase inhibitors, or both from the data. Correlations for κ are reduced, correlations for Bliss and CI are increased.

Like the compounds in the simulations described above, the compounds and cell lines in the OPPS exhibit a wide range of dose-response behaviors, so the dataset provides an excellent real-world test of the predictions from our simulated experiments (see Tables S2 and S3 for drugs/mechanistic class definitions and cell line annotations). In Fig. 1C–E, the degree of Bliss bias appears to be driven by the product of the two individual Hill slopes; to test this in the Merck dataset and compare across metrics, we correlated the results of each analysis method with the product of each agent’s Hill slope (as estimated by individual dose-response fits). Figure 2A–C depict the relationship between the Hill slope product of a combination and the estimated synergy or antagonism of the combination according to Bliss, CI, and BRAID methods (see Tables S4–S6 for all interaction metrics). As predicted by our simulations, Bliss tends towards antagonism in low-slope combinations and synergy in high-slope combinations, with a Pearson correlation coefficient of 0.57. CI similarly shows a bias towards antagonism at low slopes, albeit with a smaller correlation of 0.36. The BRAID metric κ exhibits the lowest magnitude of correlation (0.17), but *in the opposite direction*, estimating synergy at low Hill slopes and antagonism at higher slopes. This difference is critical, because one of the mechanistic classes with the lowest average Hill slope – inhibitors of the PI3K/AKT/mTOR pathway (‘mTOR Pathway’) – also exhibits the highest average synergy according to all three metrics; another low-slope class, ‘Other Kinase’ inhibitors, also exhibits above-average synergy. This suggests the correlation exhibited by κ may reflect true inter-mechanistic variations in pharmacology and interaction. Indeed, removing these classes from the dataset reduces the correlation exhibited by κ by over 46%, while actually *increasing* the biases exhibited by Bliss and CI (Fig. 2D,E). In contrast, removing two mechanistic classes with average Hill slopes (Microtubule agents and Topoisomerase inhibitors) has little or no effect on any metric.

### A real-world test for methods to analyze drug combinations

**Figure 3:**
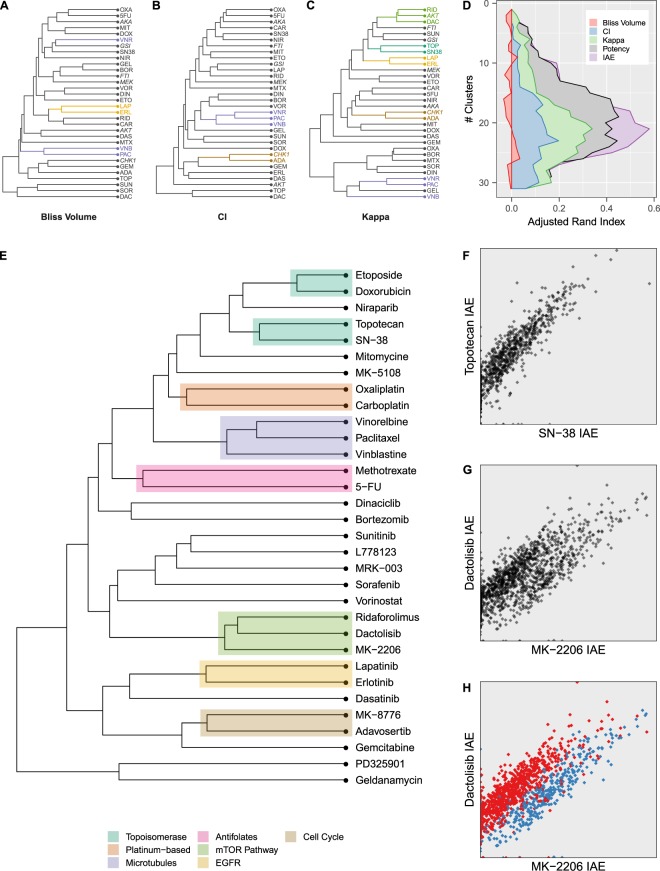
(**A–C**) Hierarchical clustering of active compounds based on interaction profile similarity using Bliss, CI, and BRAID κ. **(D)** Agreement (measured by adjusted Rand index) between the existing mechanistic classification and the clusterings of compounds at each clustering level based on all three interaction metrics, potency (as measured by log-transformed IC50 values) and IAE (also log-transformed). **(E)** Hierarchical clustering of active compounds based on combined efficacy as measured by IAE. **(F)** Stripe plot of IAE values for combination experiments containing the active metabolite of irinotecan, SN-38, against the corresponding IAE values for experiments containing Topotecan. **(G)** Stripe plot of the AKT inhibitor MK-2206 and the dual PI3K/mTOR inhibitor Dactolisib. **(H)** The same stripe plot with AKT-resistant and AKT-sensitive cell lines highlighted in red and blue, respectively.

One advantage of a well-studied dataset like the OPPS is that we can move past simple evaluations of stability and bias, and on to investigations of real-world informative content. We postulate that the type and degree of interaction (synergy, antagonism, or additivity) present in a drug combination is a function of the mechanism of action of each drug. Therefore, a combination metric that accurately gauges interaction should also be able to cluster compounds by their mechanism of action. Since the compounds in the OPPS are well-studied, this data set affords a powerful means of comparing different drug combination metrics. We applied the Bliss, CI, and BRAID methods to the 22,410 drug combinations reported in the OPPS. For hierarchical clustering, six compounds that failed to show significant activity in any model – ABT-888 (Veliparib), Cyclophosphamide, Dexamethasone, Metformin, MK-4541, and Temozolomide – were excluded, as no interaction could be determined. Figure 3A–C depict the results of hierarchical clusterings of the 32 active compounds in the dataset according to their patterns of interaction, as determined by all three combination metrics. While clustering by Bliss identifies 2 mechanistic pairs, and clustering by CI identifies two clusters containing five compounds, clustering by the BRAID metric κ identifies five mechanistic clusters containing eleven compounds. Quantitative evaluation of the clusterings confirms that the BRAID κ more closely agrees with the existing mechanistic classification than Bliss and CI (Fig. 3D).

An alternative approach to inferring compound mechanism of action is to compare the potency of a compound across a diverse panel of cell lines, as exemplified by the NCI COMPARE algorithm^24^. While most combination experiments might be limited in the scope of models tested, the OPPS – which assayed 38 drugs against 39 cancer cell lines – was comprehensive enough to define a signature of drug responses that could be used to classify compounds by their mechanism of action. Indeed, clustering by single-agent potency alone outperformed all combination analysis metrics. However, an advantage of BRAID is that it models the full two-dimensional response surface using an equation that depends on the κ metric. The BRAID index of achievable efficacy (IAE) is essentially a surface integral over this modeled drug response surface that summarizes the drug combination as a single combined efficacy. Clustering compounds according to their variations in IAE outperforms all metrics (including potency) and identifies eight mechanistic clusters (Topoisomerases, Platinum-based agents, Microtubule agents, Anti-folates, PI3K/AKT/mTOR pathway inhibitors, EGFR inhibitors, and Cell Cycle Checkpoint agents) containing eighteen compounds (Fig. 3D,E).

### Insights from the Merck data set using BRAID: PI3K/AKT/mTOR inhibitors

As encouraging as the successes of BRAID-based clustering are, equally interesting are the circumstances where it diverges from expectation. While the PI3K/AKT/mTOR inhibitors showed high levels of correlation according to interaction, forming one of the tightest κ-based clusters, their IAE-based similarity was well below several other classes. Examining specific pairwise relationships reveals the reason. Figure 3F depicts the correlation between the IAE values of SN-38 and Topotecan (both Topoisomerase I inhibitors) combinations, exhibiting a strong correlation of 0.87. Figure 3G depicts the same plot for the AKT-inhibitor MK-2206, and Dactolisib (BEZ-235), a dual PI3K/mTOR inhibitor^25,26^. Although AKT is a direct downstream target of Dactolisib, the correlation between the two drugs is noticeably lower (0.77), and examination of the plot of their IAE values appears to show two distinct stripes of activity. Highlighting a subset of eleven cell lines reveals that these stripes correspond to distinct subpopulations of cells, one of which is selectively more sensitive to combinations involving the AKT inhibitor, with efficacy increased roughly three-fold (Fig. 3H). As a further check, we analyzed this variation in relative drug combination efficacy against gene expression values reported in the Genomics of Drug Sensitivity in Cancer expression library^27^. Of the eleven genes (out of a total 17,737) whose expression related most strongly with the measured difference in IAE – each exhibiting a strength of effect over seven standard deviations from the mean – eight have been reported as modulators of AKT (Supplementary Fig. 1S and Table S7).

### Insights from the Merck data set using BRAID: targeting BRCA1-deficiency

**Figure 4:**
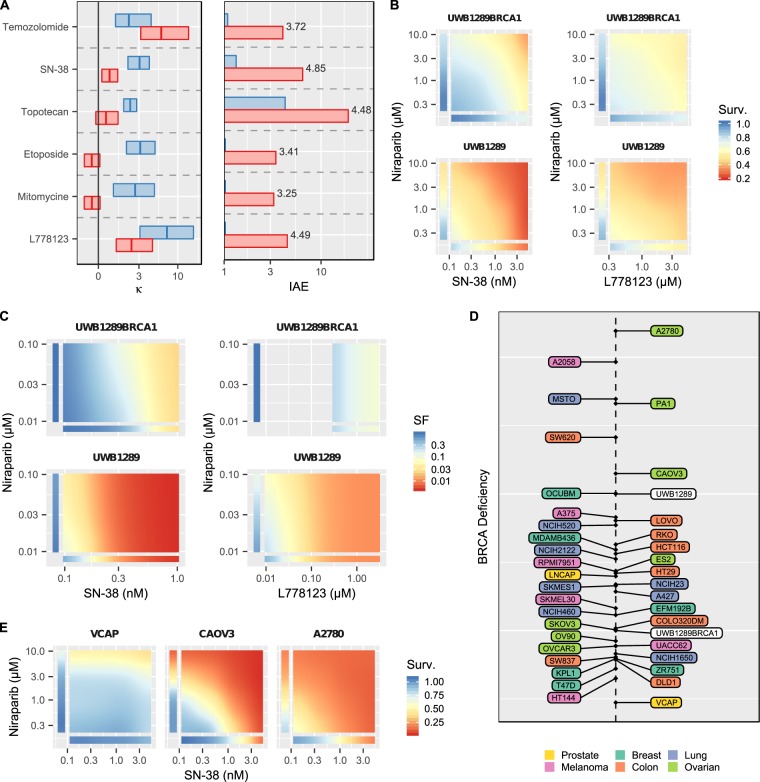
(**A**) Best fit κ values and IAE values for several drugs combined with Niraparib in the BRCA1-deficient UWB1.289 (red) and the isogenic BRCA1-restored UWB1.289+BRCA1 (blue). In the left panel, rectangles represent the estimated 95% confidence intervals on κ, and vertical crossbars represent the estimated value of κ. In the right panel, numerical labels represent the ratio of estimated IAE in UWB1.289 to IAE in UWB1.289+BRCA1. **(B)** Best fit response surfaces for OPPS experiments testing SN-38 and the prenylation inhibitor L778123 with Niraparib in both cell lines. **(C)** Best fit response surfaces from clonogenic experiments testing SN-38 and L778123 with Niraparib in both cell lines. **(D)** Projection of all Niraparib IAE values for all 39 cell lines in the OPSS onto the change in IAE between UWB1.289 and UWB1.289+BRCA1, a phenotypic measure of BRCA1 deficiency. **(E)** Best fit response surfaces for SN-38 with Niraparib for three representative cell lines at three points along the BRCA1-deficiency dimension, demonstrating a range of sensitivity to the combination.

The OPPS included UWB1.289, an ovarian cancer cell line with a loss of BRCA1 function, and UW1.289 + BRCA1, a cell line engineered from UWB1.289 to restore wild-type BRCA1 function^28^. Examination of the differences between these two isogenic cell lines allows us to gauge the impact of BRCA1 loss on the efficacy and interaction of a given drug combination. Cancer cells deficient in BRCA1 or BRCA2 are sensitive to PARP inhibitors^29^. Moreover, the potentiation between PARP inhibitors and Temozolomide is well-documented and has been or is being evaluated in several different indications in multiple clinical trials (e.g., NCT02049593, NCT01085422, NCT01139970, NCT01051596, NCT03749187)^30^. BRAID analysis of the OPPS shows that while the combination of the PARP inhibitor Niraparib and Temozolomide exhibits moderate synergy in UWB1.289 + BRCA1 (blue, κ = 1.98), this synergy is increased in BRCA1-deficient UWB1.289 (red, κ = 6.26), resulting in a nearly four-fold increase in combined efficacy (Fig. 4A). This increase in synergy, however, is not replicated in other DNA-damaging agents. In contrast, the Topoisomerase inhibitors SN-38, Topotecan, and Etoposide all exhibit moderate synergy with Niraparib in UWB1.289 + BRCA1, but this synergy is actually *reduced* in UWB1.289. Nevertheless, each of these three combinations shows a marked increase in combined efficacy, driven not by an increase in synergy, but by a shift in potency of both Niraparib and the Topoisomerase inhibitors in response to the loss of BRCA1 function. Several other compounds induced a three-fold or greater increase in combined efficacy with Niraparib, including Mitomycine, the Aurora Kinase A inhibitor MK-5108, and surprisingly, the prenylation inhibitor L778123 (Fig. 4B). Using the colony forming assay, we independently confirmed the shift in potency and augmented combined efficacy of Niraparib/SN-38 and Niraparib/L778132 in the two isogenic cell lines (Fig. 4C).

Next, we used BRAID analysis to compare the other cell lines in the OPPS to the drug combination sensitivity pattern induced by BRCA1-defiency. By projecting the behavior of all Niraparib combinations (as represented by IAE) onto the difference in behavior caused by loss of BRCA1 function (defined by the comparison between UWB1.289 and UWB1.289 + BRCA1), each cell-line can be plotted on a phenotypic dimension that captures the signature induced by BRCA1-defiency (Fig. 4D). Several cell lines, such as COLO320DM (colon cancer) and SKOV3 (ovarian), cluster near UWB1.289 + BRCA1 and demonstrate a drug sensitivity signature similar to the BRCA1-restored cell line, whereas OCUBM (breast cancer) and CAOV3 (ovarian) behave like BRCA1-deficient UWB1.289. It is interesting to note that the ovarian cell lines A2780 and PA1 lie even further along the projected dimension, suggesting they represent a “hyper-deficient” phenotype, even though both are reported to be wild-type for BRCA1 and BRCA2^31^. Examination of the response surfaces of the Niraparib/SN-38 combination in three cell lines shows close agreement with the broader projection (Fig. 4E).

## Discussion

Analysis of combined action is a question to which researchers are understandably eager to find a concrete, broadly applicable answer. Yet with a challenge so varied and complex, any solution must inevitably confront tradeoffs. In their 1995 review, Greco, Bravo, and Parsons refer to the URSA model as “*a* model, not *the* model”^22^; we feel that this level of caution is essential to a successful analysis approach. Even Loewe additivity, considered by many to be the gold standard of non-interaction, and the basis of the combination index, the interaction index, the URSA method and many others, is on tenuous theoretical ground, itself being based on the intuition that a compound cannot interact with itself. But is it not conceivable that a compound can target multiple mechanisms that enhance or hinder each other’s function? Rather than an unassailable law, we feel it is better to view Loewe additivity, and the more basic non-self-interaction principle on which it and the BRAID model are based, as easily identifiable landmarks that allow researchers to navigate the wildly variable space of combined responses, and provide a largely universal reference point against which different responses can be described and compared. Paraphrasing Box, then, the question is not “is our model wrong?”, but “is our model useful?”

Though the intuitions underlying the methods are sensible, simulations and real-world data sets demonstrate that the two most common methods to analyze drug combination, Bliss and CI, have instability and biases that are difficult or even impossible to eliminate. Further, in the case of the more robust implementation of CI, the complex circumstances underlying the differing biases make it equally challenging to even predict when and where they will occur. The BRAID model, by comparison, shows a greater and more consistent level of stability, and no discernible bias across a range of conditions, suggesting that it possesses greater potential to make useful predictions. To test that potential, however, real-world data is needed.

**Figure 5:**
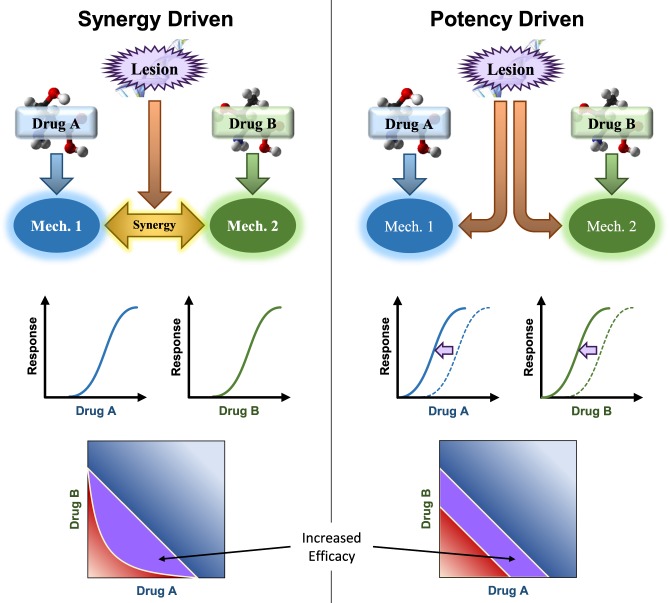
Schematic representation of synergy-driven vs. potency-driven combination efficacy. In synergy-driven efficacy, a sample condition such as a genetic lesion drives a distinct mechanism that increases the synergy between mechanism 1 and mechanism 2. The effects of drug A and drug B are unaffected by the lesion, but their combined effect is increased, and a set of combination dose pairs that would not have produced a sufficient efficacy now do; the combination is more effective. In potency-driven efficacy, the lesion simply increases the sensitivity to mechanisms 1 and 2 simultaneously, causing both drugs to be more potent. The degree of synergy is unchanged, but again the combination shows greater efficacy.

Evaluation of the Merck OPPS demonstrates the power of a robust combination evaluation method. Not only did the interaction parameter κ reveal more of the mechanistic structure present than either Bliss or CI, the IAE, a more robust surface metric dependent on κ and an explicit surface model, was able to illuminate nearly all compound mechanisms present, as well as unexpected behaviors such as the selective efficacy of the AKT inhibitor MK-2206. Deeper evaluation of Niraparib combinations revealed a complex space of responses, in which synergistic interaction and combined efficacy can vary independently of one another. This fact has helped us appreciate two forms of combination efficacy, illustrated in Fig. 5. While the first, synergy-driven efficacy, attracts the lion’s share of attention in translational research, the second form, potency-driven efficacy, exhibited by Niraparib combined with SN-38 or L778123, can be just as important to therapeutic development. Only the first class of efficacy, however, can be identified by methods that distill analysis down to a single index of synergy or antagonism, meaning that reliance on such methods can force a broad spectrum of promising combination therapies out of the development pipeline.

Equally important to the usefulness of our model is that it be accessible to as many researchers as possible. In our first BRAID paper^17^, we released open-source analysis and visualization packages, “braidrm” and “braidReports”, for the R statistical computing environment^32^. Using these R packages, we have also built a free-to-use web application at http://braid.stjude.org/webapp/ that enables users to upload their own data sets to run BRAID analyses. Finally, to enable further discovery, we have published a fully interactive web visualization of our analysis of the Merck OPPS at http://braid.stjude.org/mercktour/, in which users can view overall patterns of interaction and combined efficacy, build stripe plots for any pair of drugs, and view and interrogate individual combination experiments.

## Methods

### Additive surface simulation

All simulated response surfaces were defined by seven parameters, the selection of which is described in later sections. These seven parameters are:

- *ID*_*M,A*_: The dose of median effect of the first compound; the concentration at which the dose-response function for drug *A* produces an effect halfway between its minimal effect and its maximal effect
- *ID*_*M,B*_: The dose of median effect of the second compound
- *n*_*a*_: The (positive) Hill slope of the first drug as defined in a standard log-logistic function
- *n*_*b*_: The Hill slope of the second drug
- *E*_0_: The observed effect or measure when no drug is present
- *E*_*f,A*_: The maximal effect of the first compound; the observed effect when very large concentrations of drug *A* are present in isolation
- *E*_*f,B*_: The maximal effect of the second compound

To avoid biasing results towards our own model, additive surfaces were not generated using the BRAID model, but instead using an implementation of Loewe additivity described by Greco, Park & Rustum; effectively, we implemented their URSA (universal response surface approach) model, but fixed the interaction parameter α to 0. However, as this model does not allow the maximal effects of the two drugs to differ, we extended the traditional definition of Loewe additivity to a more general property called *asymptotic* additivity.

The definition of asymptotic additivity is as follows: if one of the two drugs (assume without loss of generality that it is drug B) has a “smaller” maximal effect (meaning a smaller difference between initial and maximal effects) in isolation, then each true dose of drug B is transformed in to an “effective dose”, which is the dose that would produce the same effect if drug B had identical dose response parameters but the same maximal effect as drug A. Mathematically, this is expressed as:

$${D{\prime} }_{B}=I{D}_{M,B}{\left(\frac{\left(\frac{{E}_{f,B}-{E}_{0}}{{E}_{f,A}-{E}_{0}}\right){\left(\frac{{D}_{B}}{I{D}_{M,B}}\right)}^{{n}_{b}}}{1+\left(1-\frac{{E}_{f,B}-{E}_{0}}{{E}_{f,A}-{E}_{0}}\right){\left(\frac{{D}_{B}}{I{D}_{M,B}}\right)}^{{n}_{b}}}\right)}^{\frac{1}{{n}_{b}}}$$

This effective dose of drug B and the dose of drug A are then input into the Loewe additive response surface model with *E*_*f,A*_ as the overall maximal effect. Simulated surfaces were also subject to random noise; the actual measured value at a given dose-pair was simulated by adding a normally-distributed random value (with the standard deviation specified below) to the modeled additive surface.

### Bliss relative bias simulations

Because Bliss Volume does not have established thresholds for synergy and antagonism, our Bliss volume simulations simulated an entire combination screen across multiple agents. Specifically, eight “compounds” were screened in an all against all arrangement. All compounds had doses of median effect at one 1 μM, with dose response curves ranging from 0% effect to 100% effect. The only difference between the eight compounds was the Hill slope, which ranged from 0.5 to 4, in 0.5 increments. The dose response of a compound was simulated at 15 concentrations in a two-fold dilution, centered on 1 μM; the combined effects of two compounds was simulated in a 10-by-10 checkerboard, with concentrations also in a two-fold dilution, with a maximum of 32 μM. All studies were simulated with noise with a standard deviation of 5%.

For each simulated screen, the simulation began by setting the thresholds for synergy and antagonism by examining the distribution of self-vs.-self combinations. This proceeded as follows: for each of the “compounds”, the following steps were performed three times.

1. The dose response of the “compound” was simulated twice (once as drug A and once as drug B)
2. The combined effect of the drug (with itself) was simulated in a checker board fashion.
3. The Bliss volume for the combined surface was estimated from the individual dose response curves and the combined response surface

Performing this procedure resulted in 24 measurements of self-vs.-self Bliss Volumes across all compounds, and allowed for the estimation of the 2.5 percentile and 97.5 percentile, which were then used as thresholds for antagonism and synergy respectively.

Armed with simulated thresholds, the simulated screen proceeded to an all-vs-all stage. The individual dose response of all eight compound was again tested, once as drug A and once as drug B, and the combined effects of all 64 combinations (including self-vs.-self) were tested. Bliss Volumes were then estimated for all 64 two-compound combinations, which constituted the results of the simulated screen. From simulation to simulation, a count was made of the number of simulations in which a given two-drug combination was judged antagonistic, additive, or synergistic. A total of 1000 simulated screens were run.

### Combination index bias simulation

Because synergy and antagonism thresholds on the Combination Index are better established, the simulations evaluating bias in the combination index were limited to simulating single combination studies. For each simulated experiment, each drug was tested in dose-response at 15 concentrations in a two-fold dilution centered on 1 μM; the two drugs were then tested in combination in a checkerboard of dose pairs, with 10 different combinations of both drugs, also in a two-fold dilution with a maximum of 32 μM. Within simulated experiment, all measurements were simulated in triplicate. All experiments were simulated with Gaussian noise with a standard deviation of 7.5%. In addition, all concentrations were simulated as varying around the intended target concentration in a log normal distribution, such that the standard deviation of the natural logarithm of the simulated concentration was 0.953 (the natural logarithm of 1.1). Unless otherwise specified, compounds had doses of median effect of 1 μM, Hill slopes of 1, initial effects of 0%, and maximal effects of 100%.

Blocks of simulated experiments were broken into three groups, each intended to evaluate the impact of a different pharmacological condition: varying (but equal) Hill slopes, inversely varying Hill slopes, and differing maximal effects. In the first group, simulations were run with both drugs possessing Hill slopes of 2, 3, and 4, in addition to the default value of 1. In the second group, simulations were run in which had differing Hill slopes: 1 and 1.5, for a ratio of 1.5; 0.9 and 1.8, for a ratio of 2; 0.8 and 2.4, for a ratio of 3; and 0.75 and 3, for a ratio of 4. In the third group, the maximal effect of the second drug was lowered to values of 90%, 80%, 70% and 60%. For each set of pharmacological conditions, 1000 simulated experiments were run, to determine the likelihood that an estimate of synergy or antagonism would result from a simulated additive surface.

Dose response curves were fit to data in one of two ways. The first, used to calculate what we call the “Chou Index,” is the median-effect method of Chou and Talalay, in which dose response data are linearized by passing them through a logit transform, and then fit using standard linear least squares regression. Because this transform can only be applied to values within the range 0% to 100%, all values below 0.5% effect and 99.5% effect were clipped to these values. The resulting linear fit can then be transformed back to produce a standard log-logistic Hill curve. The second method, which we refer to simply as the “Combination Index,” uses non-linear least-squares optimization to fit a log-logistic curve directly to the measured values. Because the Combination Index requires compounds to have equal bounds on effect, all curves were constrained to initial and maximal effects of 0% and 100%, respectively. Nonlinear optimization was performed using the “optim” function in the “stats” package in the R Statistical Computing Environment.

For each simulated experiment, the dose response parameters, IC50, and IC99 were estimated for both individual compounds, as well as for the seven longest diagonals of the combined checkerboard (representing seven constant-ratio combinations ranging from 8:1 to 1:8). The IC50 and IC99 values were compared to estimate the combination index values for each of these seven constant-ratio combinations at the 50% and 99% effect level. These combination index values were saved across all 1000 iterations, to give an estimate of what proportion of constant ratio combinations would be judged synergistic or antagonistic for that set of pharmacological conditions. A combination was judged antagonistic at a particular level if the combination index at that level was above 2 (representing an decrease in potency of two-fold or more relative to individual agents), and judged synergistic if the combination index was below 0.5 (representing an increase in potency of two-fold or more).

For comparison, the BRAID response surface method was run on the same simulated surfaces used to evaluate the Chou Index and Combination Index. Each surface was fit using the “findBestBRAID” function of the “braidrm” R package (version 0.72) in the R Statistical Computing Environment. Surfaces were judged as antagonistic if the confidence interval on the interaction parameter κ lay entirely below 0; they were judged synergistic if the confidence interval lay entirely above 0.

### Merck oncopolypharmacology screen metric estimation

Data from the Merck OncoPolyPharmacology Screen (OPPS) were obtained from the supplemental data from the paper by O’Neil *et al*. All published measurements were represented as fractional viability, so no additional normalization was required. All combinations in the OPPS were fit using the “braidrm” function in “braidrm” R package (version 0.72) using the standard eight-parameter model; this ensured that all combinations were fully fit with all eight parameters to facilitate comparison between surfaces. IAE values were estimated using the highest concentration tested in combination for each drug as upper limits, and 50% survival as the threshold for efficacy. For all combinations, individual dose-response curves were fit using the “optim” non-linear optimization function in the “stats” R package, with the initial and maximal effects set at 1 and 0, respectively. Bliss volume was estimated by taking the average difference between the measured survival and the predicted Bliss independent surfaces based on the individual dose-response curves. Combination index values were estimated by fitting the dose-response of the diagonal of the 4-by-4 combination checkerboard, and calculating combination index from the estimated IC50 values for all three dose response curves.

Additionally, all individual dose response curves were fit using the “findBestHill” function in the the “braidrm” package (with 1 and 0 as default initial and maximal effects). These fits were used to estimate the Hill slope and potency of each drug in each cell-line. Potency was measured as the ratio of the highest concentration tested to the concentration (if any) that produced a viability of 0.5; if this level was not reached within the range tested, the potency was set at a minimum value of 1.

### Merck oncopolypharmacology screen clustering

When one compound exhibits little activity, it is difficult to determine the nature or degree of interaction, so interaction values tend to be very unstable. These instabilities can significantly disrupt a hierarchical clustering, so six compounds which exhibited less than 25% killing on average at their highest concentrations were excluded from clustering analyses (Table S2). To estimate the similarity between two compounds based on a given interaction measure, all combinations containing the first drug were matched with all combinations of the second drug such that the tested cell line and partner drug were the same (partner drug/cell line combinations that were tested in with one drug but not the other were excluded for that similarity calculation). This produced two large vectors of metric values representing the “interaction profiles” of those compounds; similarity between interaction measure profiles was estimated using cosine similarity, as this way of measuring similarity emphasize the sign of the values as well as their changes. Similarity based on therapeutic index values (for potency) was estimated in the same way, except values were log-transformed and similarity was determined using Pearson’s correlation coefficient. Finally, similarity for IAE values was estimated by calculating Pearson’s correlation coefficient, and then subtracting the mean correlation between the activity of each compound and the activity of compounds in isolation (the *null-compound* correlation). This adjustment was introduced after we observed that largely inactive compounds exhibited misleadingly high similarities: for inactive compounds, combination activity is driven entirely by the partner compound, so inactive compounds exhibit highly similar patterns of combined efficacy. Hierarchical clustering based on a similarity matrix was performed by treating each compound as a trivial cluster, and then iteratively combining the two remaining clusters such that the average similarity between compounds in each cluster is maximized. This was repeated until only one cluster remained.

Because the hierarchical clustering proceeds in a pairwise iterative fashion, it also imposes 32 different partitions of increasing coarseness on the set of 32 compounds, ranging from the trivial 32-piece partition in which every compound is its own cluster to the trivial 1-piece partition in which all compounds are in a single cluster. Comparing these 32 partitions with the existing mechanistic classification using the adjusted Rand index allows us to quantify the agreement between the clustering and compound mechanism across many scales, and the average of the adjusted Rand index values gives an effective measure of overall agreement between the hierarchical clustering and compound mechanism.

### Genomic analysis of AKT sensitivity

As in the estimation of inter-compound similarity, all combination experiments involving the AKT inhibitor MK-2206 were matched with the corresponding experiments with the same cell-line and partner drug, but the PI3K/mTOR inhibitor Dactolisib instead of MK-2206. These pairs of experiments were broken down by cell line and the average change between the base-10 logarithm of the IAE50 value for the combination involving MK-2206 and the base-10 logarithm of the IAE50 value for the combination involving Dactolisib was estimated for all cell-lines. This value was used as an estimate of *relative AKT sensitivity* for that cell line. For most cell lines, the value was around 0.5 indicating that combinations with Dactolisib had roughly 3-times higher IAE50 values than those involving MK-2206 in those lines. However, for the AKT sensitive cell lines, the relative AKT sensitivity is clustered around 0, indicating MK-2206 combinations were just as potent as Dactolisib combinations. For cell lines present in both the Merck Oncopolypharmacology Screen and the GDSC expression database (32 cell lines), expression values for 17,737 genes were correlated with the measure of relative AKT sensitivity, and the eleven genes with the largest linear coefficient were selected, meaning those genes that saw the largest change in expression (in either direction) from the less AKT-sensitive cell lines to the more AKT-sensitive cell lines. Using the magnitude of the linear relationship avoided false positives in which small noisy variations in expression exhibit randomly high correlations with our measure of interest.

### Clonogenic evaluation of drug combinations

Human ovarian carcinoma cells UWB1.289 (BRCA1-null) and UWB1.289 BRCA1 (BRCA1 wild-type function restored) were purchased from American Type Culture Collection (Manassas, Virginia) and cultured according to supplier recommendations. Cells were plated in 6-well Corning plates (Corning, New York) with 250 cells per well in 3 mL of media and incubated overnight in a 5% CO_2_ incubator at 37 °C. Test compounds were prepared and serially diluted in DMSO and media. The next day media was carefully aspirated from each well and fresh media with vehicle or drug was added. Treated cells were incubated for 8 to 10 days for colonies to develop. Colonies were fixed and stained. Media was briefly aspirated and 2 ml per well of 4% paraformaldehyde (Electron Microscopy Services, Hatfield, PA) prepared in phosphate buffered saline was added for 10 minutes at room temperature. The paraformaldehyde solution was aspirated and 2 ml per well of a filtered 0.05% crystal violet solution prepared in distilled water was added. Cells were stained for 30 minutes at room temperature. The crystal violet was removed and plates were washed carefully by immersion in distilled water, using a 4 L beaker, gently rinsing until the water ran clear. Plates were then placed face down over their lids to dry overnight at room temperature. Plates were finally imaged at 1600dpi resolution using an Epson V700 scanner. Colonies were quantified using ImageJ.

Given the count of colonies for all combined doses of two drugs, the BRAID equation was used as a model of plating efficiency (in logarithmic space). The model was fit using maximum likelihood estimation: the number of colonies was treated as a variable-rate Poisson process, in which the mean number of colonies observed was determined by multiplying the modeled plating efficiency (the output of the BRAID equation) by the seeding density, and BRAID surface parameters were optimized so that the log-likelihood of the observed colony counts was maximized.

## Supplementary information

Supplementary Information.

Supplementary Information2.

Supplementary Information3.
