# Supplementary material for: A critical evaluation of methods to interpret drug combinations
Source: Sci Rep. 2020 Mar 20;10:5144. doi: 10.1038/s41598-020-61923-1 (PMC7083968; doi:10.1038/s41598-020-61923-1)
Supplement: Supplementary file 2 — Supplementary Information2. [file 41598_2020_61923_MOESM2_ESM.docx]

**A critical evaluation of methods to interpret drug combinations**

Nathaniel R. Twarog^1^, Michele Connelly^1^, and Anang A. Shelat^1*^

^1^Department of Chemical Biology and Therapeutics, St. Jude Children’s Research Hospital, 262 Danny Thomas Place, Memphis, TN 38105

**KEY WORDS:** drug combination, synergy, antagonism, Bliss, Combination Index, BRAID

**CORRESPONDING AUTHOR:** Dr. Anang A. Shelat

Department of Chemical Biology and Therapeutics,

St. Jude Children’s Research Hospital,

262 Danny Thomas Place, MS1000

Memphis, TN 38105

Email: [anang.shelat@stjude.org](mailto:anang.shelat@stjude.org)

T: 901.595.5751

F: 901.595.5715

**Supplemental Table and Figure legends**

Supplemental Table S1. | Bliss thresholds from simulated self-self combinations

Supplemental Table S2. | OPPS drug list

Supplemental Table S3. | OPPS cell lines

Supplemental Table S4. | Interaction metrics calculated for each drug combination in each cell line in the OPPS data set

Supplemental Table S5. | Average interaction metrics for each drug combination across all cell lines in the OPPS data set

Supplemental Table S6. | Single-agent curve fit parameters for each drug in each cell line in the OPPS data set

Supplemental Table S7. | Genes correlated with AKT sensitivity

Supplemental Figure S1. | Volcano plot to identify genes correlated with sensitivity to the AKT inhibitor MK-2206 vs. the dual PI3K/mTOR inhibitor Dactolisib.
