# Supplementary figures and images for: A critical evaluation of methods to interpret drug combinations
Source: Sci Rep. 2020 Mar 20;10:5144. doi: 10.1038/s41598-020-61923-1 (PMC7083968; doi:10.1038/s41598-020-61923-1)

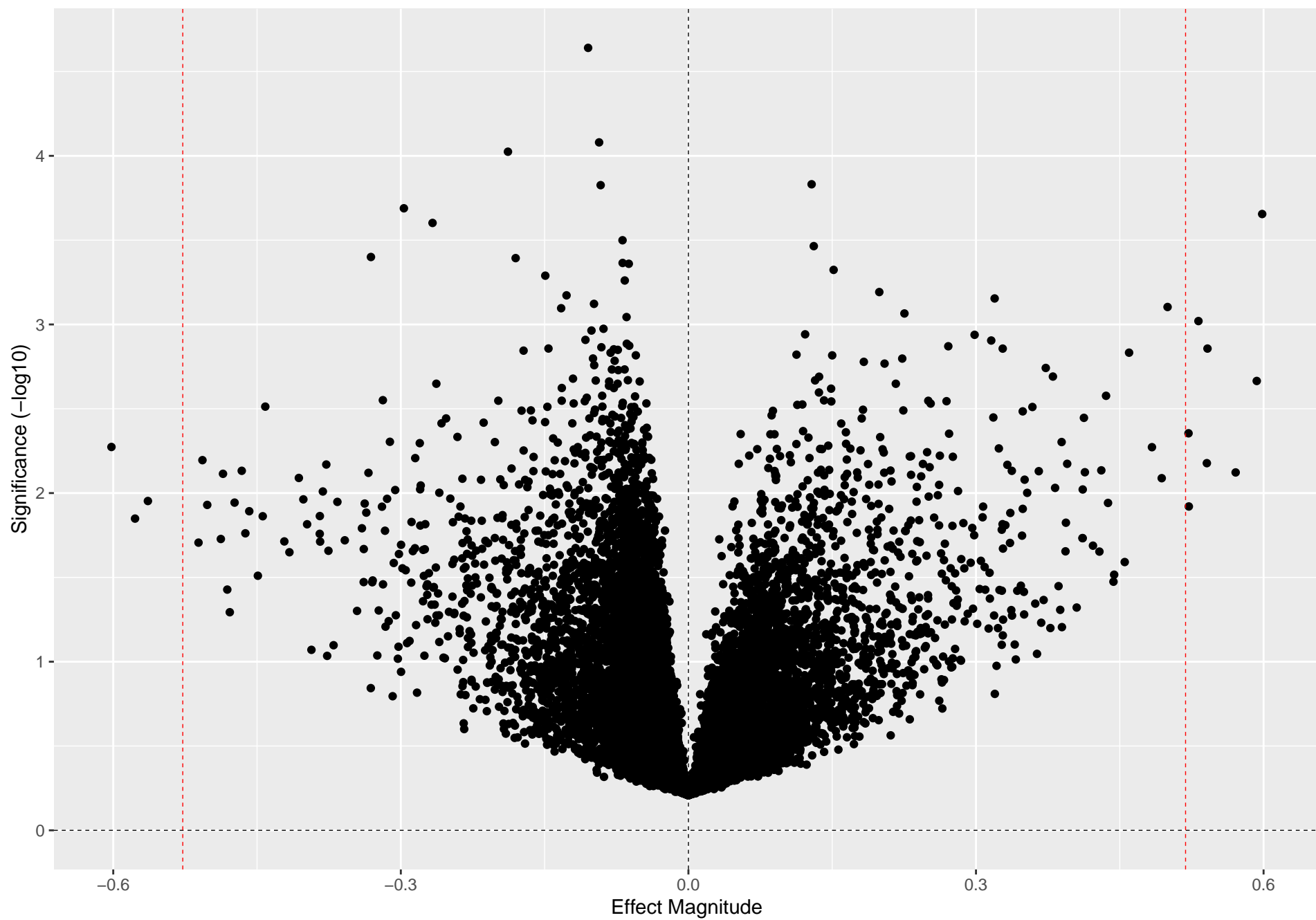

Supplement: Supplementary file 3 — Supplementary Information3. [file 41598_2020_61923_MOESM3_ESM.pdf]
